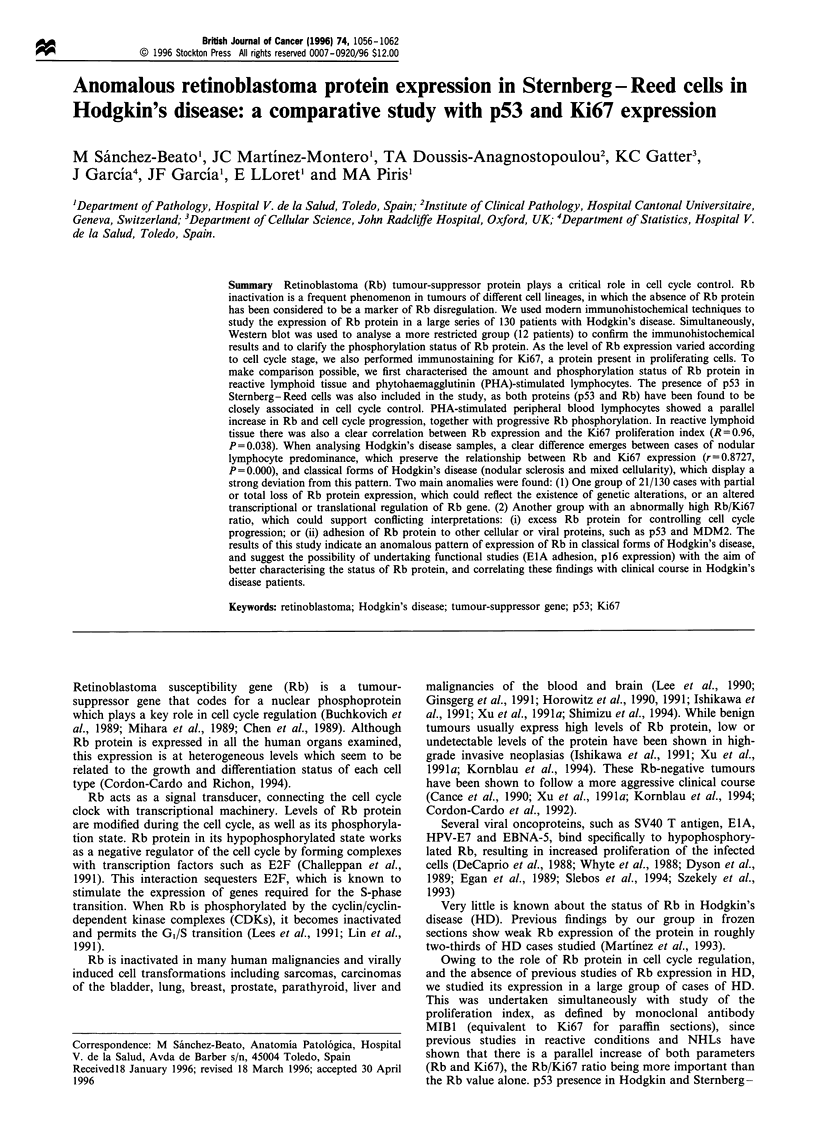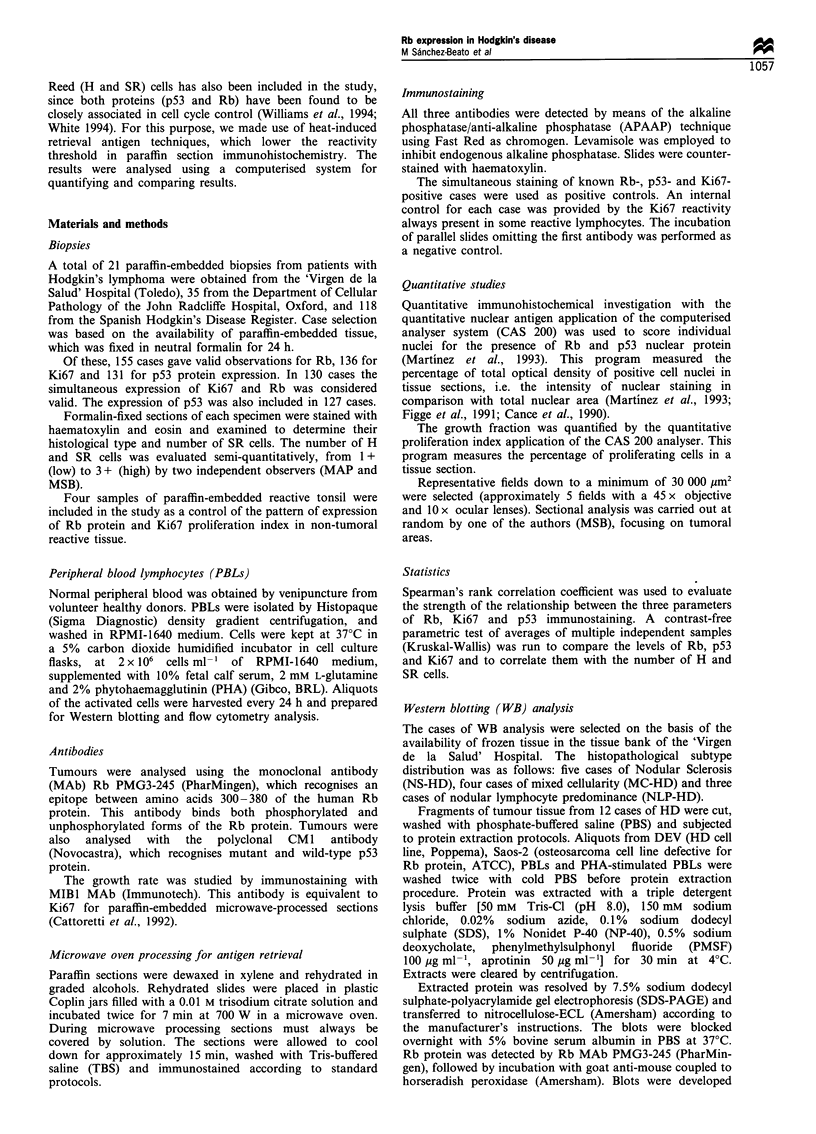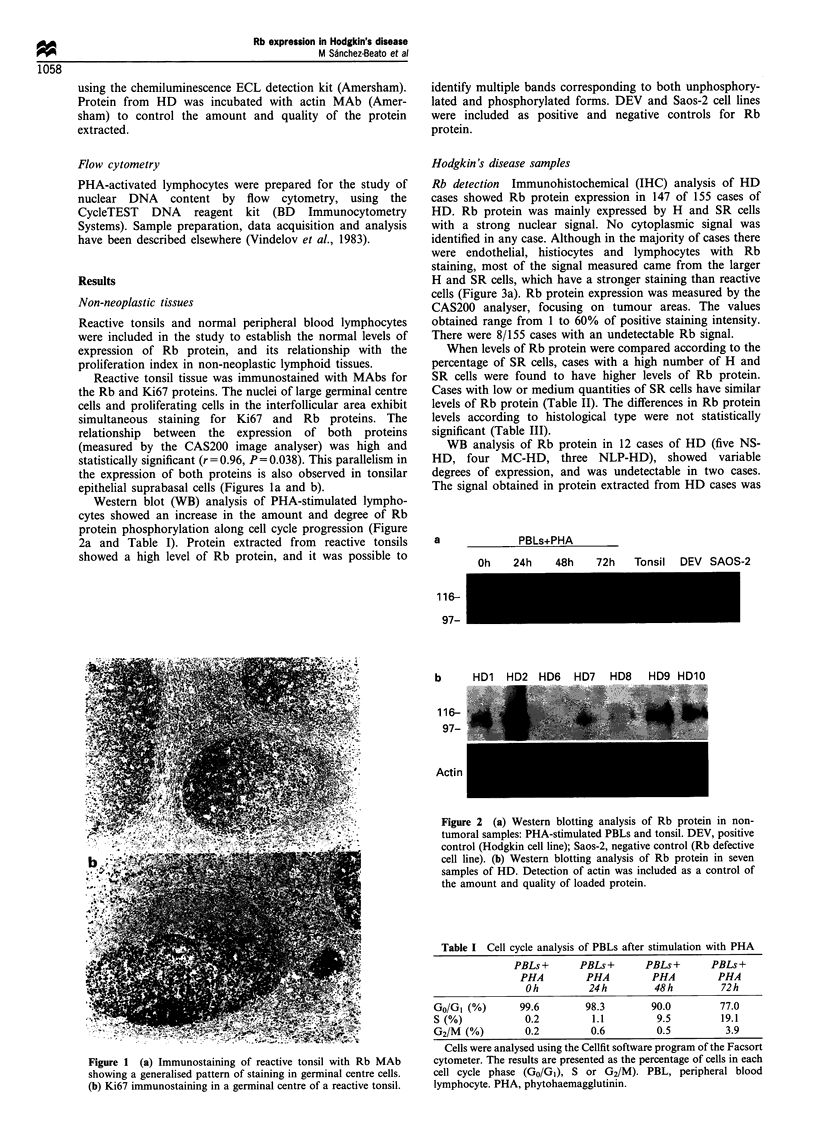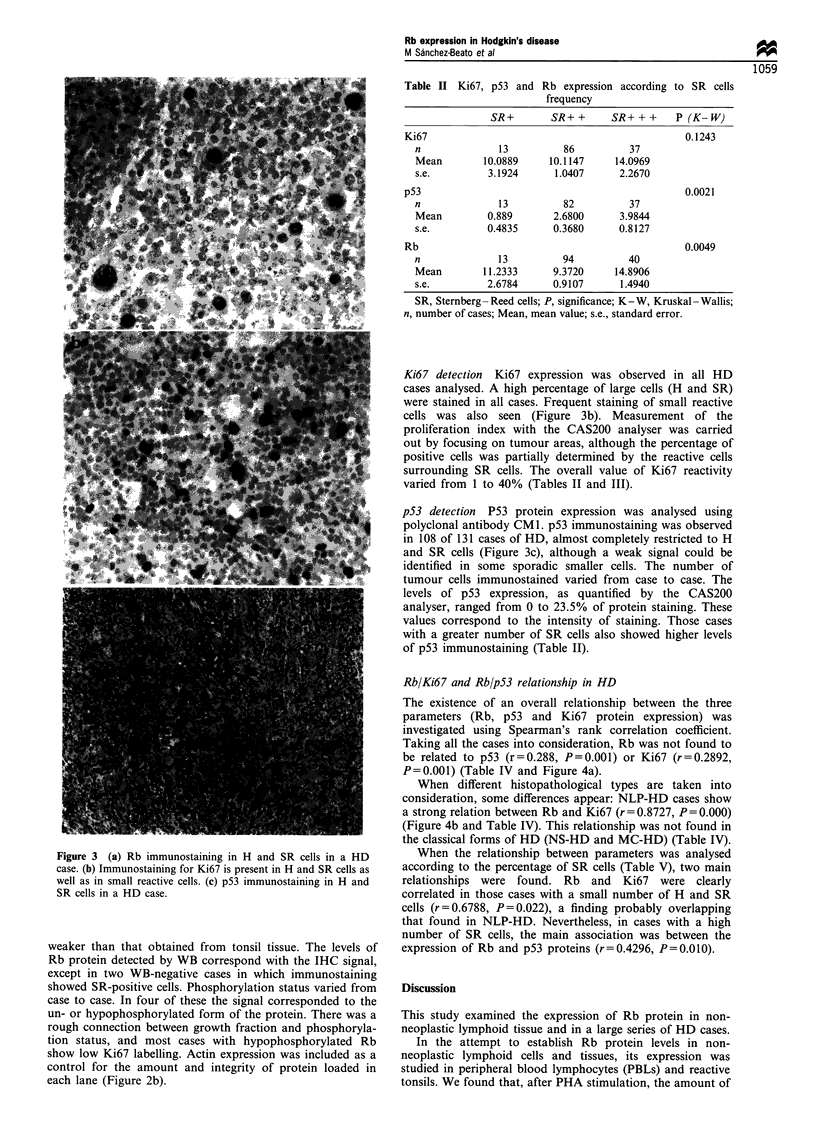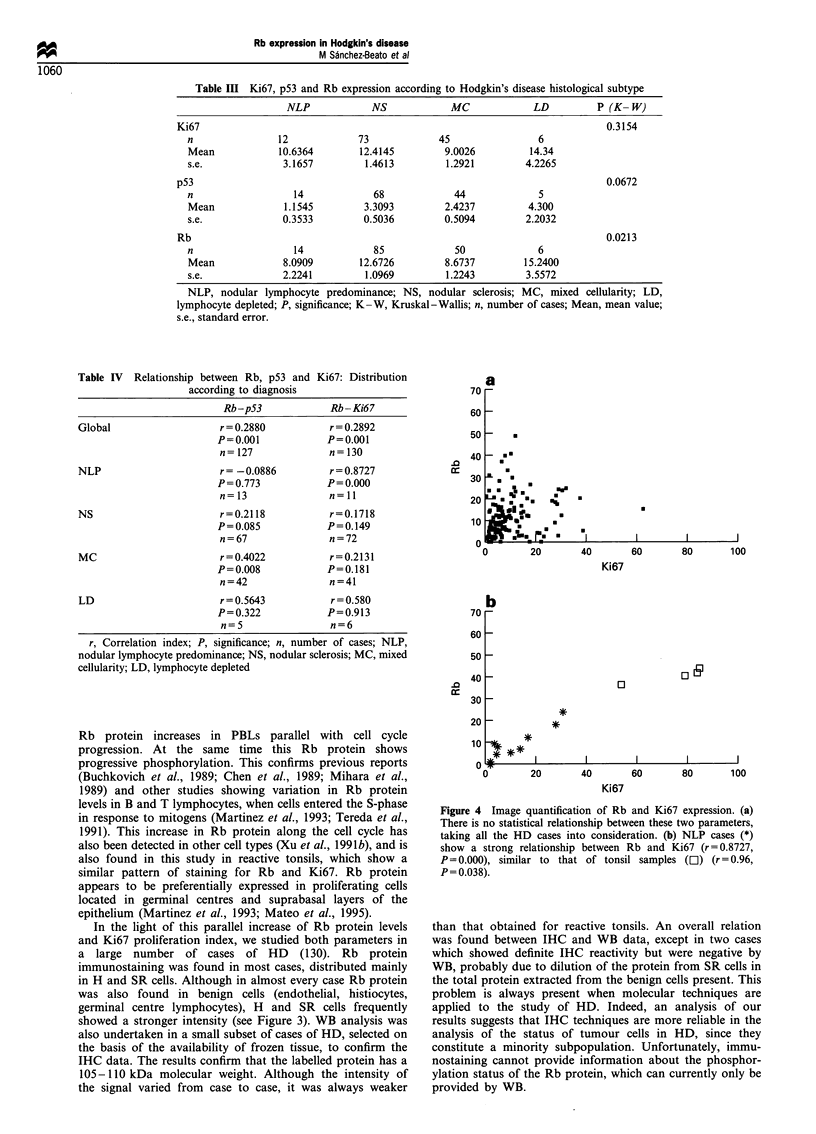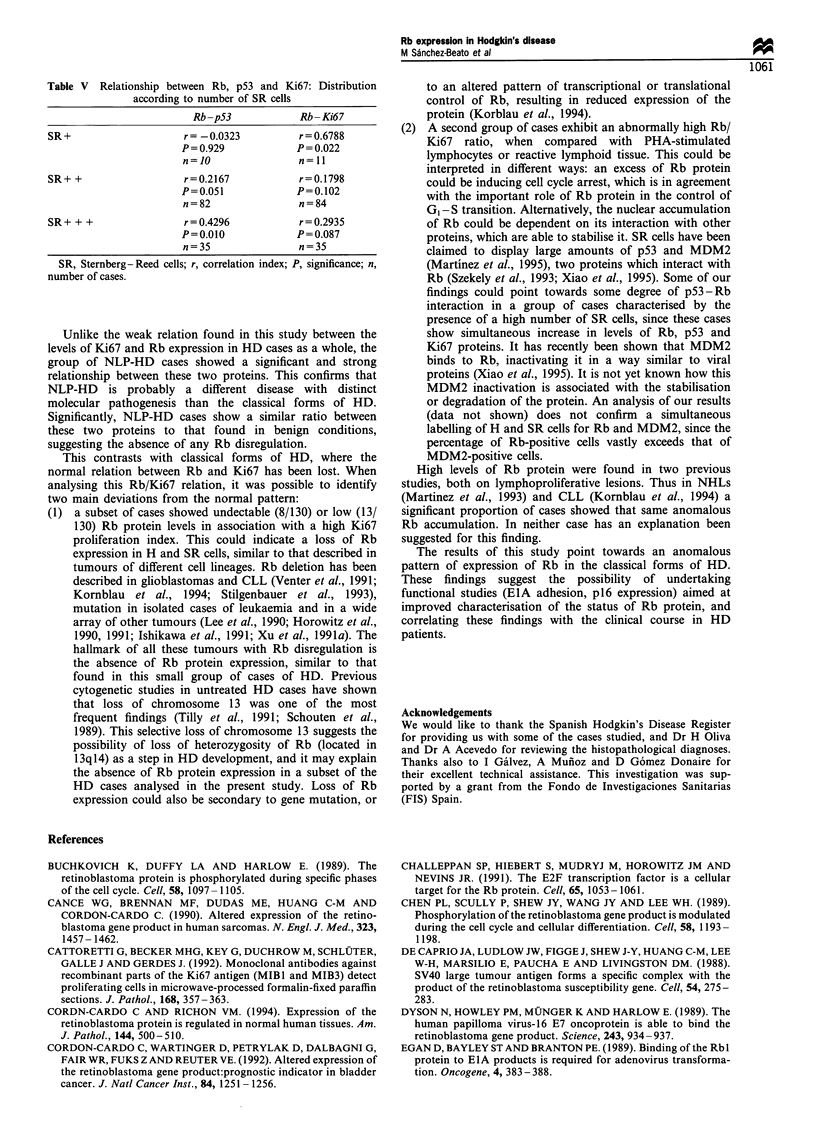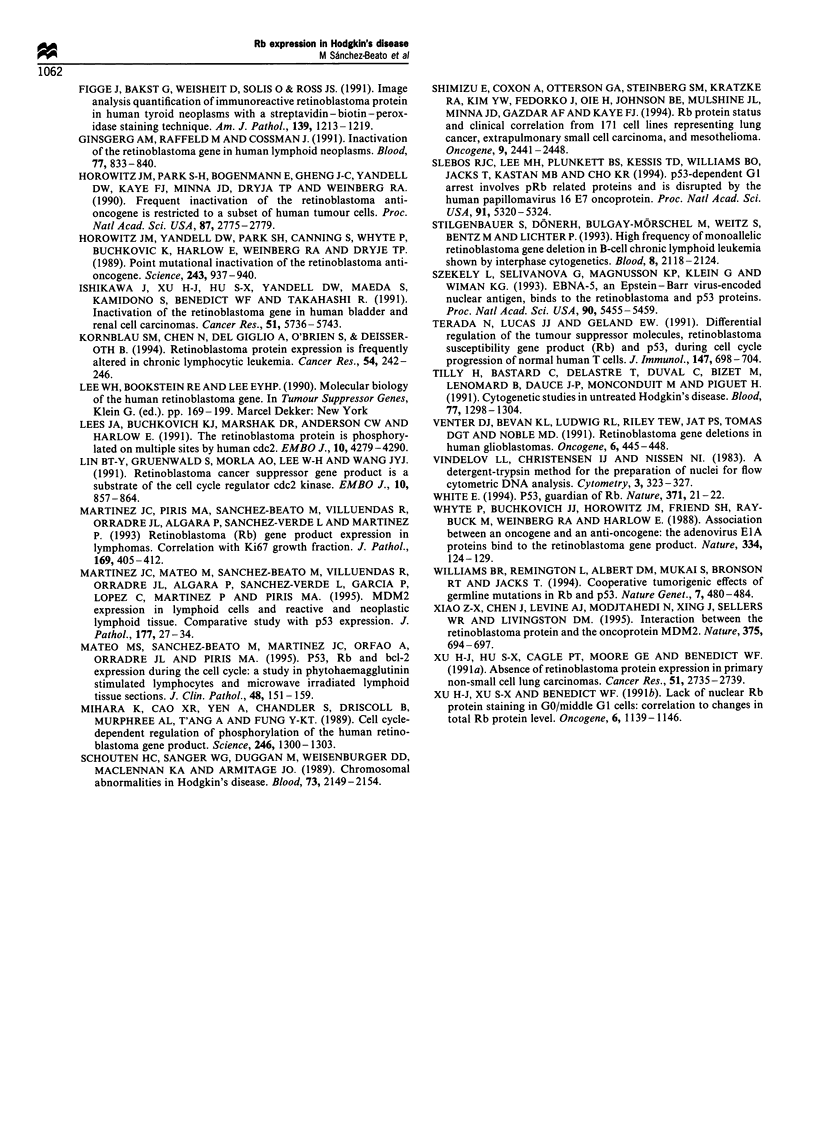# Anomalous retinoblastoma protein expression in Sternberg-Reed cells in Hodgkin's disease: a comparative study with p53 and Ki67 expression.

**DOI:** 10.1038/bjc.1996.489

**Published:** 1996-10

**Authors:** M. Sánchez-Beato, J. C. Martínez-Montero, T. A. Doussis-Anagnostopoulou, K. C. Gatter, J. García, J. F. García, E. LLoret, M. A. Piris

**Affiliations:** Department of Pathology, Hospital V. de la Salud, Toledo, Spain.

## Abstract

**Images:**


British Journal of Cancer (1996) 74, 1056-1062
© 1996 Stockton Press All rights reserved 0007-0920/96 $12.00

### Anomalous retinoblastoma protein expression in Sternberg-Reed cells in Hodgkin's disease: a comparative study with p53 and Ki67 expression

M Sánchez-Beato[1], JC Martínez-Montero[1], TA Doussis-Anagnostopoulou[2], KC Gatter[3], J García[4], JF García[1], E LLoret[1] and MA Piris[1]

[1]*Department of Pathology, Hospital V. de la Salud, Toledo, Spain;* [2]*Institute of Clinical Pathology, Hospital Cantonal Universitaire, Geneva, Switzerland;* [3]*Department of Cellular Science, John Radcliffe Hospital, Oxford, UK;* [4]*Department of Statistics, Hospital V. de la Salud, Toledo, Spain.*

**Summary** Retinoblastoma (Rb) tumour-suppressor protein plays a critical role in cell cycle control. Rb inactivation is a frequent phenomenon in tumours of different cell lineages, in which the absence of Rb protein has been considered to be a marker of Rb disregulation. We used modern immunohistochemical techniques to study the expression of Rb protein in a large series of 130 patients with Hodgkin's disease. Simultaneously, Western blot was used to analyse a more restricted group (12 patients) to confirm the immunohistochemical results and to clarify the phosphorylation status of Rb protein. As the level of Rb expression varied according to cell cycle stage, we also performed immunostaining for Ki67, a protein present in proliferating cells. To make comparison possible, we first characterised the amount and phosphorylation status of Rb protein in reactive lymphoid tissue and phytohaemagglutinin (PHA)-stimulated lymphocytes. The presence of p53 in Sternberg-Reed cells was also included in the study, as both proteins (p53 and Rb) have been found to be closely associated in cell cycle control. PHA-stimulated peripheral blood lymphocytes showed a parallel increase in Rb and cell cycle progression, together with progressive Rb phosphorylation. In reactive lymphoid tissue there was also a clear correlation between Rb expression and the Ki67 proliferation index ($R=0.96$, $P=0.038$). When analysing Hodgkin's disease samples, a clear difference emerges between cases of nodular lymphocyte predominance, which preserve the relationship between Rb and Ki67 expression ($r=0.8727$, $P=0.000$), and classical forms of Hodgkin's disease (nodular sclerosis and mixed cellularity), which display a strong deviation from this pattern. Two main anomalies were found: (1) One group of 21/130 cases with partial or total loss of Rb protein expression, which could reflect the existence of genetic alterations, or an altered transcriptional or translational regulation of Rb gene. (2) Another group with an abnormally high Rb/Ki67 ratio, which could support conflicting interpretations: (i) excess Rb protein for controlling cell cycle progression; or (ii) adhesion of Rb protein to other cellular or viral proteins, such as p53 and MDM2. The results of this study indicate an anomalous pattern of expression of Rb in classical forms of Hodgkin's disease, and suggest the possibility of undertaking functional studies (E1A adhesion, p16 expression) with the aim of better characterising the status of Rb protein, and correlating these findings with clinical course in Hodgkin's disease patients.

**Keywords:** retinoblastoma; Hodgkin's disease; tumour-suppressor gene; p53; Ki67

Retinoblastoma susceptibility gene (Rb) is a tumour-suppressor gene that codes for a nuclear phosphoprotein which plays a key role in cell cycle regulation (Buchkovich *et al.*, 1989; Mihara *et al.*, 1989; Chen *et al.*, 1989). Although Rb protein is expressed in all the human organs examined, this expression is at heterogeneous levels which seem to be related to the growth and differentiation status of each cell type (Cordon-Cardo and Richon, 1994).

Rb acts as a signal transducer, connecting the cell cycle clock with transcriptional machinery. Levels of Rb protein are modified during the cell cycle, as well as its phosphorylation state. Rb protein in its hypophosphorylated state works as a negative regulator of the cell cycle by forming complexes with transcription factors such as E2F (Challeppan *et al.*, 1991). This interaction sequesters E2F, which is known to stimulate the expression of genes required for the S-phase transition. When Rb is phosphorylated by the cyclin/cyclin-dependent kinase complexes (CDKs), it becomes inactivated and permits the $G_1/S$ transition (Lees *et al.*, 1991; Lin *et al.*, 1991).

Rb is inactivated in many human malignancies and virally induced cell transformations including sarcomas, carcinomas of the bladder, lung, breast, prostate, parathyroid, liver and malignancies of the blood and brain (Lee *et al.*, 1990; Ginsgerg *et al.*, 1991; Horowitz *et al.*, 1990, 1991; Ishikawa *et al.*, 1991; Xu *et al.*, 1991*a*; Shimizu *et al.*, 1994). While benign tumours usually express high levels of Rb protein, low or undetectable levels of the protein have been shown in high-grade invasive neoplasias (Ishikawa *et al.*, 1991; Xu *et al.*, 1991*a*; Kornblau *et al.*, 1994). These Rb-negative tumours have been shown to follow a more aggressive clinical course (Cance *et al.*, 1990; Xu *et al.*, 1991*a*; Kornblau *et al.*, 1994; Cordon-Cardo *et al.*, 1992).

Several viral oncoproteins, such as SV40 T antigen, E1A, HPV-E7 and EBNA-5, bind specifically to hypophosphorylated Rb, resulting in increased proliferation of the infected cells (DeCaprio *et al.*, 1988; Whyte *et al.*, 1988; Dyson *et al.*, 1989; Egan *et al.*, 1989; Slebos *et al.*, 1994; Szekely *et al.*, 1993)

Very little is known about the status of Rb in Hodgkin's disease (HD). Previous findings by our group in frozen sections show weak Rb expression of the protein in roughly two-thirds of HD cases studied (Martínez *et al.*, 1993).

Owing to the role of Rb protein in cell cycle regulation, and the absence of previous studies of Rb expression in HD, we studied its expression in a large group of cases of HD. This was undertaken simultaneously with study of the proliferation index, as defined by monoclonal antibody MIB1 (equivalent to Ki67 for paraffin sections), since previous studies in reactive conditions and NHLs have shown that there is a parallel increase of both parameters (Rb and Ki67), the Rb/Ki67 ratio being more important than the Rb value alone. p53 presence in Hodgkin and Sternberg-

Correspondence: M Sánchez-Beato, Anatomía Patológica, Hospital V. de la Salud, Avda de Barber s/n, 45004 Toledo, Spain
Received18 January 1996; revised 18 March 1996; accepted 30 April 1996

Reed (H and SR) cells has also been included in the study, since both proteins (p53 and Rb) have been found to be closely associated in cell cycle control (Williams *et al.*, 1994; White 1994). For this purpose, we made use of heat-induced retrieval antigen techniques, which lower the reactivity threshold in paraffin section immunohistochemistry. The results were analysed using a computerised system for quantifying and comparing results.

### Materials and methods

### *Biopsies*

A total of 21 paraffin-embedded biopsies from patients with Hodgkin's lymphoma were obtained from the 'Virgen de la Salud' Hospital (Toledo), 35 from the Department of Cellular Pathology of the John Radcliffe Hospital, Oxford, and 118 from the Spanish Hodgkin's Disease Register. Case selection was based on the availability of paraffin-embedded tissue, which was fixed in neutral formalin for 24 h.

Of these, 155 cases gave valid observations for Rb, 136 for Ki67 and 131 for p53 protein expression. In 130 cases the simultaneous expression of Ki67 and Rb was considered valid. The expression of p53 was also included in 127 cases.

Formalin-fixed sections of each specimen were stained with haematoxylin and eosin and examined to determine their histological type and number of SR cells. The number of H and SR cells was evaluated semi-quantitatively, from 1+ (low) to 3+ (high) by two independent observers (MAP and MSB).

Four samples of paraffin-embedded reactive tonsil were included in the study as a control of the pattern of expression of Rb protein and Ki67 proliferation index in non-tumoral reactive tissue.

### *Peripheral blood lymphocytes (PBLs)*

Normal peripheral blood was obtained by venipuncture from volunteer healthy donors. PBLs were isolated by Histopaque (Sigma Diagnostic) density gradient centrifugation, and washed in RPMI-1640 medium. Cells were kept at 37°C in a 5% carbon dioxide humidified incubator in cell culture flasks, at $2\times10^6$ cells $ml^{-1}$ of RPMI-1640 medium, supplemented with 10% fetal calf serum, 2 mM L-glutamine and 2% phytohaemagglutinin (PHA) (Gibco, BRL). Aliquots of the activated cells were harvested every 24 h and prepared for Western blotting and flow cytometry analysis.

### *Antibodies*

Tumours were analysed using the monoclonal antibody (MAb) Rb PMG3-245 (PharMingen), which recognises an epitope between amino acids 300–380 of the human Rb protein. This antibody binds both phosphorylated and unphosphorylated forms of the Rb protein. Tumours were also analysed with the polyclonal CM1 antibody (Novocastra), which recognises mutant and wild-type p53 protein.

The growth rate was studied by immunostaining with MIB1 MAb (Immunotech). This antibody is equivalent to Ki67 for paraffin-embedded microwave-processed sections (Cattoretti *et al.*, 1992).

### *Microwave oven processing for antigen retrieval*

Paraffin sections were dewaxed in xylene and rehydrated in graded alcohols. Rehydrated slides were placed in plastic Coplin jars filled with a 0.01 M trisodium citrate solution and incubated twice for 7 min at 700 W in a microwave oven. During microwave processing sections must always be covered by solution. The sections were allowed to cool down for approximately 15 min, washed with Tris-buffered saline (TBS) and immunostained according to standard protocols.

### *Immunostaining*

All three antibodies were detected by means of the alkaline phosphatase/anti-alkaline phosphatase (APAAP) technique using Fast Red as chromogen. Levamisole was employed to inhibit endogenous alkaline phosphatase. Slides were counter-stained with haematoxylin.

The simultaneous staining of known Rb-, p53- and Ki67-positive cases were used as positive controls. An internal control for each case was provided by the Ki67 reactivity always present in some reactive lymphocytes. The incubation of parallel slides omitting the first antibody was performed as a negative control.

### *Quantitative studies*

Quantitative immunohistochemical investigation with the quantitative nuclear antigen application of the computerised analyser system (CAS 200) was used to score individual nuclei for the presence of Rb and p53 nuclear protein (Martínez *et al.*, 1993). This program measured the percentage of total optical density of positive cell nuclei in tissue sections, i.e. the intensity of nuclear staining in comparison with total nuclear area (Martínez *et al.*, 1993; Figge *et al.*, 1991; Cance *et al.*, 1990).

The growth fraction was quantified by the quantitative proliferation index application of the CAS 200 analyser. This program measures the percentage of proliferating cells in a tissue section.

Representative fields down to a minimum of 30 000 $\mu m^2$ were selected (approximately 5 fields with a 45× objective and 10× ocular lenses). Sectional analysis was carried out at random by one of the authors (MSB), focusing on tumoral areas.

### *Statistics*

Spearman's rank correlation coefficient was used to evaluate the strength of the relationship between the three parameters of Rb, Ki67 and p53 immunostaining. A contrast-free parametric test of averages of multiple independent samples (Kruskal-Wallis) was run to compare the levels of Rb, p53 and Ki67 and to correlate them with the number of H and SR cells.

### *Western blotting (WB) analysis*

The cases of WB analysis were selected on the basis of the availability of frozen tissue in the tissue bank of the 'Virgen de la Salud' Hospital. The histopathological subtype distribution was as follows: five cases of Nodular Sclerosis (NS-HD), four cases of mixed cellularity (MC-HD) and three cases of nodular lymphocyte predominance (NLP-HD).

Fragments of tumour tissue from 12 cases of HD were cut, washed with phosphate-buffered saline (PBS) and subjected to protein extraction protocols. Aliquots from DEV (HD cell line, Poppema), Saos-2 (osteosarcoma cell line defective for Rb protein, ATCC), PBLs and PHA-stimulated PBLs were washed twice with cold PBS before protein extraction procedure. Protein was extracted with a triple detergent lysis buffer [50 mM Tris-Cl (pH 8.0), 150 mM sodium chloride, 0.02% sodium azide, 0.1% sodium dodecyl sulphate (SDS), 1% Nonidet P-40 (NP-40), 0.5% sodium deoxycholate, phenylmethylsulphonyl fluoride (PMSF) 100 $\mu g\ ml^{-1}$, aprotinin 50 $\mu g\ ml^{-1}$] for 30 min at 4°C. Extracts were cleared by centrifugation.

Extracted protein was resolved by 7.5% sodium dodecyl sulphate-polyacrylamide gel electrophoresis (SDS-PAGE) and transferred to nitrocellulose-ECL (Amersham) according to the manufacturer's instructions. The blots were blocked overnight with 5% bovine serum albumin in PBS at 37°C. Rb protein was detected by Rb MAb PMG3-245 (PharMingen), followed by incubation with goat anti-mouse coupled to horseradish peroxidase (Amersham). Blots were developed using the chemiluminescence ECL detection kit (Amersham). Protein from HD was incubated with actin MAb (Amersham) to control the amount and quality of the protein extracted.

*Flow cytometry*

PHA-activated lymphocytes were prepared for the study of nuclear DNA content by flow cytometry, using the CycleTEST DNA reagent kit (BD Immunocytometry Systems). Sample preparation, data acquisition and analysis have been described elsewhere (Vindelov *et al.*, 1983).

### Results

*Non-neoplastic tissues*

Reactive tonsils and normal peripheral blood lymphocytes were included in the study to establish the normal levels of expression of Rb protein, and its relationship with the proliferation index in non-neoplastic lymphoid tissues.

Reactive tonsil tissue was immunostained with MAbs for the Rb and Ki67 proteins. The nuclei of large germinal centre cells and proliferating cells in the interfollicular area exhibit simultaneous staining for Ki67 and Rb proteins. The relationship between the expression of both proteins (measured by the CAS200 image analyser) was high and statistically significant ($r=0.96$, $P=0.038$). This parallelism in the expression of both proteins is also observed in tonsilar epithelial suprabasal cells (Figures 1a and b).

Western blot (WB) analysis of PHA-stimulated lymphocytes showed an increase in the amount and degree of Rb protein phosphorylation along cell cycle progression (Figure 2a and Table I). Protein extracted from reactive tonsils showed a high level of Rb protein, and it was possible to identify multiple bands corresponding to both unphosphorylated and phosphorylated forms. DEV and Saos-2 cell lines were included as positive and negative controls for Rb protein.

*Hodgkin's disease samples*

*Rb detection* Immunohistochemical (IHC) analysis of HD cases showed Rb protein expression in 147 of 155 cases of HD. Rb protein was mainly expressed by H and SR cells with a strong nuclear signal. No cytoplasmic signal was identified in any case. Although in the majority of cases there were endothelial, histiocytes and lymphocytes with Rb staining, most of the signal measured came from the larger H and SR cells, which have a stronger staining than reactive cells (Figure 3a). Rb protein expression was measured by the CAS200 analyser, focusing on tumour areas. The values obtained range from 1 to 60% of positive staining intensity. There were 8/155 cases with an undetectable Rb signal.

When levels of Rb protein were compared according to the percentage of SR cells, cases with a high number of H and SR cells were found to have higher levels of Rb protein. Cases with low or medium quantities of SR cells have similar levels of Rb protein (Table II). The differences in Rb protein levels according to histological type were not statistically significant (Table III).

WB analysis of Rb protein in 12 cases of HD (five NS-HD, four MC-HD, three NLP-HD), showed variable degrees of expression, and was undetectable in two cases. The signal obtained in protein extracted from HD cases was

**Figure 1** (**a**) Immunostaining of reactive tonsil with Rb MAb showing a generalised pattern of staining in germinal centre cells. (**b**) Ki67 immunostaining in a germinal centre of a reactive tonsil.

**Figure 2** (**a**) Western blotting analysis of Rb protein in non-tumoral samples: PHA-stimulated PBLs and tonsil. DEV, positive control (Hodgkin cell line); Saos-2, negative control (Rb defective cell line). (**b**) Western blotting analysis of Rb protein in seven samples of HD. Detection of actin was included as a control of the amount and quality of loaded protein.

**Table I** Cell cycle analysis of PBLs after stimulation with PHA

| | *PBLs+ PHA 0 h* | *PBLs+ PHA 24 h* | *PBLs+ PHA 48 h* | *PBLs+ PHA 72 h* |
|---|---|---|---|---|
| $G_0/G_1$ (%) | 99.6 | 98.3 | 90.0 | 77.0 |
| S (%) | 0.2 | 1.1 | 9.5 | 19.1 |
| $G_2/M$ (%) | 0.2 | 0.6 | 0.5 | 3.9 |

Cells were analysed using the Cellfit software program of the Facsort cytometer. The results are presented as the percentage of cells in each cell cycle phase ($G_0/G_1$), S or $G_2/M$). PBL, peripheral blood lymphocyte. PHA, phytohaemagglutinin.

**Figure 3** (**a**) Rb immunostaining in H and SR cells in a HD case. (**b**) Immunostaining for Ki67 is present in H and SR cells as well as in small reactive cells. (**c**) p53 immunostaining in H and SR cells in a HD case.

weaker than that obtained from tonsil tissue. The levels of Rb protein detected by WB correspond with the IHC signal, except in two WB-negative cases in which immunostaining showed SR-positive cells. Phosphorylation status varied from case to case. In four of these the signal corresponded to the un- or hypophosphorylated form of the protein. There was a rough connection between growth fraction and phosphorylation status, and most cases with hypophosphorylated Rb show low Ki67 labelling. Actin expression was included as a control for the amount and integrity of protein loaded in each lane (Figure 2b).

**Table II** Ki67, p53 and Rb expression according to SR cells frequency

| | *SR+* | *SR+ +* | *SR+ + +* | P *(K−W)* |
|---|---|---|---|---|
| Ki67 | | | | 0.1243 |
| *n* | 13 | 86 | 37 | |
| Mean | 10.0889 | 10.1147 | 14.0969 | |
| s.e. | 3.1924 | 1.0407 | 2.2670 | |
| p53 | | | | 0.0021 |
| *n* | 13 | 82 | 37 | |
| Mean | 0.889 | 2.6800 | 3.9844 | |
| s.e. | 0.4835 | 0.3680 | 0.8127 | |
| Rb | | | | 0.0049 |
| *n* | 13 | 94 | 40 | |
| Mean | 11.2333 | 9.3720 | 14.8906 | |
| s.e. | 2.6784 | 0.9107 | 1.4940 | |

SR, Sternberg−Reed cells; *P*, significance; K−W, Kruskal−Wallis; *n*, number of cases; Mean, mean value; s.e., standard error.

*Ki67 detection* Ki67 expression was observed in all HD cases analysed. A high percentage of large cells (H and SR) were stained in all cases. Frequent staining of small reactive cells was also seen (Figure 3b). Measurement of the proliferation index with the CAS200 analyser was carried out by focusing on tumour areas, although the percentage of positive cells was partially determined by the reactive cells surrounding SR cells. The overall value of Ki67 reactivity varied from 1 to 40% (Tables II and III).

*p53 detection* P53 protein expression was analysed using polyclonal antibody CM1. p53 immunostaining was observed in 108 of 131 cases of HD, almost completely restricted to H and SR cells (Figure 3c), although a weak signal could be identified in some sporadic smaller cells. The number of tumour cells immunostained varied from case to case. The levels of p53 expression, as quantified by the CAS200 analyser, ranged from 0 to 23.5% of protein staining. These values correspond to the intensity of staining. Those cases with a greater number of SR cells also showed higher levels of p53 immunostaining (Table II).

### *Rb/Ki67 and Rb/p53 relationship in HD*

The existence of an overall relationship between the three parameters (Rb, p53 and Ki67 protein expression) was investigated using Spearman's rank correlation coefficient. Taking all the cases into consideration, Rb was not found to be related to p53 ($r=0.288$, $P=0.001$) or Ki67 ($r=0.2892$, $P=0.001$) (Table IV and Figure 4a).

When different histopathological types are taken into consideration, some differences appear: NLP-HD cases show a strong relation between Rb and Ki67 ($r=0.8727$, $P=0.000$) (Figure 4b and Table IV). This relationship was not found in the classical forms of HD (NS-HD and MC-HD) (Table IV).

When the relationship between parameters was analysed according to the percentage of SR cells (Table V), two main relationships were found. Rb and Ki67 were clearly correlated in those cases with a small number of H and SR cells ($r=0.6788$, $P=0.022$), a finding probably overlapping that found in NLP-HD. Nevertheless, in cases with a high number of SR cells, the main association was between the expression of Rb and p53 proteins ($r=0.4296$, $P=0.010$).

### Discussion

This study examined the expression of Rb protein in non-neoplastic lymphoid tissue and in a large series of HD cases.

In the attempt to establish Rb protein levels in non-neoplastic lymphoid cells and tissues, its expression was studied in peripheral blood lymphocytes (PBLs) and reactive tonsils. We found that, after PHA stimulation, the amount of

**Table III** Ki67, p53 and Rb expression according to Hodgkin's disease histological subtype

| | *NLP* | *NS* | *MC* | *LD* | P *(K-W)* |
|---|---|---|---|---|---|
| Ki67 | | | | | 0.3154 |
| *n* | 12 | 73 | 45 | 6 | |
| Mean | 10.6364 | 12.4145 | 9.0026 | 14.34 | |
| s.e. | 3.1657 | 1.4613 | 1.2921 | 4.2265 | |
| p53 | | | | | 0.0672 |
| *n* | 14 | 68 | 44 | 5 | |
| Mean | 1.1545 | 3.3093 | 2.4237 | 4.300 | |
| s.e. | 0.3533 | 0.5036 | 0.5094 | 2.2032 | |
| Rb | | | | | 0.0213 |
| *n* | 14 | 85 | 50 | 6 | |
| Mean | 8.0909 | 12.6726 | 8.6737 | 15.2400 | |
| s.e. | 2.2241 | 1.0969 | 1.2243 | 3.5572 | |

NLP, nodular lymphocyte predominance; NS, nodular sclerosis; MC, mixed cellularity; LD, lymphocyte depleted; *P*, significance; K-W, Kruskal-Wallis; *n*, number of cases; Mean, mean value; s.e., standard error.

**Table IV** Relationship between Rb, p53 and Ki67: Distribution according to diagnosis

| | *Rb-p53* | *Rb-Ki67* |
|---|---|---|
| Global | $r=0.2880$<br>$P=0.001$<br>$n=127$ | $r=0.2892$<br>$P=0.001$<br>$n=130$ |
| NLP | $r=-0.0886$<br>$P=0.773$<br>$n=13$ | $r=0.8727$<br>$P=0.000$<br>$n=11$ |
| NS | $r=0.2118$<br>$P=0.085$<br>$n=67$ | $r=0.1718$<br>$P=0.149$<br>$n=72$ |
| MC | $r=0.4022$<br>$P=0.008$<br>$n=42$ | $r=0.2131$<br>$P=0.181$<br>$n=41$ |
| LD | $r=0.5643$<br>$P=0.322$<br>$n=5$ | $r=0.580$<br>$P=0.913$<br>$n=6$ |

*r*, Correlation index; *P*, significance; *n*, number of cases; NLP, nodular lymphocyte predominance; NS, nodular sclerosis; MC, mixed cellularity; LD, lymphocyte depleted

**Figure 4** Image quantification of Rb and Ki67 expression. (**a**) There is no statistical relationship between these two parameters, taking all the HD cases into consideration. (**b**) NLP cases (*) show a strong relationship between Rb and Ki67 ($r=0.8727$, $P=0.000$), similar to that of tonsil samples (□) ($r=0.96$, $P=0.038$).

Rb protein increases in PBLs parallel with cell cycle progression. At the same time this Rb protein shows progressive phosphorylation. This confirms previous reports (Buchkovich *et al.*, 1989; Chen *et al.*, 1989; Mihara *et al.*, 1989) and other studies showing variation in Rb protein levels in B and T lymphocytes, when cells entered the S-phase in response to mitogens (Martinez *et al.*, 1993; Tereda *et al.*, 1991). This increase in Rb protein along the cell cycle has also been detected in other cell types (Xu *et al.*, 1991*b*), and is also found in this study in reactive tonsils, which show a similar pattern of staining for Rb and Ki67. Rb protein appears to be preferentially expressed in proliferating cells located in germinal centres and suprabasal layers of the epithelium (Martinez *et al.*, 1993; Mateo *et al.*, 1995).

In the light of this parallel increase of Rb protein levels and Ki67 proliferation index, we studied both parameters in a large number of cases of HD (130). Rb protein immunostaining was found in most cases, distributed mainly in H and SR cells. Although in almost every case Rb protein was also found in benign cells (endothelial, histiocytes, germinal centre lymphocytes), H and SR cells frequently showed a stronger intensity (see Figure 3). WB analysis was also undertaken in a small subset of cases of HD, selected on the basis of the availability of frozen tissue, to confirm the IHC data. The results confirm that the labelled protein has a 105-110 kDa molecular weight. Although the intensity of the signal varied from case to case, it was always weaker than that obtained for reactive tonsils. An overall relation was found between IHC and WB data, except in two cases which showed definite IHC reactivity but were negative by WB, probably due to dilution of the protein from SR cells in the total protein extracted from the benign cells present. This problem is always present when molecular techniques are applied to the study of HD. Indeed, an analysis of our results suggests that IHC techniques are more reliable in the analysis of the status of tumour cells in HD, since they constitute a minority subpopulation. Unfortunately, immunostaining cannot provide information about the phosphorylation status of the Rb protein, which can currently only be provided by WB.

**Table V** Relationship between Rb, p53 and Ki67: Distribution according to number of SR cells

| | Rb−p53 | Rb−Ki67 |
|---|---|---|
| SR+ | $r = -0.0323$<br>$P = 0.929$<br>$n = 10$ | $r = 0.6788$<br>$P = 0.022$<br>$n = 11$ |
| SR++ | $r = 0.2167$<br>$P = 0.051$<br>$n = 82$ | $r = 0.1798$<br>$P = 0.102$<br>$n = 84$ |
| SR+++ | $r = 0.4296$<br>$P = 0.010$<br>$n = 35$ | $r = 0.2935$<br>$P = 0.087$<br>$n = 35$ |

SR, Sternberg−Reed cells; *r*, correlation index; *P*, significance; *n*, number of cases.

Unlike the weak relation found in this study between the levels of Ki67 and Rb expression in HD cases as a whole, the group of NLP-HD cases showed a significant and strong relationship between these two proteins. This confirms that NLP-HD is probably a different disease with distinct molecular pathogenesis than the classical forms of HD. Significantly, NLP-HD cases show a similar ratio between these two proteins to that found in benign conditions, suggesting the absence of any Rb disregulation.

This contrasts with classical forms of HD, where the normal relation between Rb and Ki67 has been lost. When analysing this Rb/Ki67 relation, it was possible to identify two main deviations from the normal pattern:

(1) a subset of cases showed undectable (8/130) or low (13/130) Rb protein levels in association with a high Ki67 proliferation index. This could indicate a loss of Rb expression in H and SR cells, similar to that described in tumours of different cell lineages. Rb deletion has been described in glioblastomas and CLL (Venter *et al.*, 1991; Kornblau *et al.*, 1994; Stilgenbauer *et al.*, 1993), mutation in isolated cases of leukaemia and in a wide array of other tumours (Lee *et al.*, 1990; Horowitz *et al.*, 1990, 1991; Ishikawa *et al.*, 1991; Xu *et al.*, 1991*a*). The hallmark of all these tumours with Rb disregulation is the absence of Rb protein expression, similar to that found in this small group of cases of HD. Previous cytogenetic studies in untreated HD cases have shown that loss of chromosome 13 was one of the most frequent findings (Tilly *et al.*, 1991; Schouten *et al.*, 1989). This selective loss of chromosome 13 suggests the possibility of loss of heterozygosity of Rb (located in 13q14) as a step in HD development, and it may explain the absence of Rb protein expression in a subset of the HD cases analysed in the present study. Loss of Rb expression could also be secondary to gene mutation, or to an altered pattern of transcriptional or translational control of Rb, resulting in reduced expression of the protein (Korblau *et al.*, 1994).

(2) A second group of cases exhibit an abnormally high Rb/Ki67 ratio, when compared with PHA-stimulated lymphocytes or reactive lymphoid tissue. This could be interpreted in different ways: an excess of Rb protein could be inducing cell cycle arrest, which is in agreement with the important role of Rb protein in the control of $G_1-S$ transition. Alternatively, the nuclear accumulation of Rb could be dependent on its interaction with other proteins, which are able to stabilise it. SR cells have been claimed to display large amounts of p53 and MDM2 (Martínez *et al.*, 1995), two proteins which interact with Rb (Szekely *et al.*, 1993; Xiao *et al.*, 1995). Some of our findings could point towards some degree of p53−Rb interaction in a group of cases characterised by the presence of a high number of SR cells, since these cases show simultaneous increase in levels of Rb, p53 and Ki67 proteins. It has recently been shown that MDM2 binds to Rb, inactivating it in a way similar to viral proteins (Xiao *et al.*, 1995). It is not yet known how this MDM2 inactivation is associated with the stabilisation or degradation of the protein. An analysis of our results (data not shown) does not confirm a simultaneous labelling of H and SR cells for Rb and MDM2, since the percentage of Rb-positive cells vastly exceeds that of MDM2-positive cells.

High levels of Rb protein were found in two previous studies, both on lymphoproliferative lesions. Thus in NHLs (Martinez *et al.*, 1993) and CLL (Kornblau *et al.*, 1994) a significant proportion of cases showed that same anomalous Rb accumulation. In neither case has an explanation been suggested for this finding.

The results of this study point towards an anomalous pattern of expression of Rb in the classical forms of HD. These findings suggest the possibility of undertaking functional studies (E1A adhesion, p16 expression) aimed at improved characterisation of the status of Rb protein, and correlating these findings with the clinical course in HD patients.

**Acknowledgements**
We would like to thank the Spanish Hodgkin's Disease Register for providing us with some of the cases studied, and Dr H Oliva and Dr A Acevedo for reviewing the histopathological diagnoses. Thanks also to I Gálvez, A Muñoz and D Gómez Donaire for their excellent technical assistance. This investigation was supported by a grant from the Fondo de Investigaciones Sanitarias (FIS) Spain.

### References

BUCHKOVICH K, DUFFY LA AND HARLOW E. (1989). The retinoblastoma protein is phosphorylated during specific phases of the cell cycle. *Cell*, **58**, 1097−1105.

CANCE WG, BRENNAN MF, DUDAS ME, HUANG C-M AND CORDON-CARDO C. (1990). Altered expression of the retinoblastoma gene product in human sarcomas. *N. Engl. J. Med.*, **323**, 1457−1462.

CATTORETTI G, BECKER MHG, KEY G, DUCHROW M, SCHLÜTER, GALLE J AND GERDES J. (1992). Monoclonal antibodies against recombinant parts of the Ki67 antigen (MIB1 and MIB3) detect proliferating cells in microwave-processed formalin-fixed paraffin sections. *J. Pathol.*, **168**, 357−363.

CORDN-CARDO C AND RICHON VM. (1994). Expression of the retinoblastoma protein is regulated in normal human tissues. *Am. J. Pathol.*, **144**, 500−510.

CORDON-CARDO C, WARTINGER D, PETRYLAK D, DALBAGNI G, FAIR WR, FUKS Z AND REUTER VE. (1992). Altered expression of the retinoblastoma gene product:prognostic indicator in bladder cancer. *J. Natl Cancer Inst.*, **84**, 1251−1256.

CHALLEPPAN SP, HIEBERT S, MUDRYJ M, HOROWITZ JM AND NEVINS JR. (1991). The E2F transcription factor is a cellular target for the Rb protein. *Cell*, **65**, 1053−1061.

CHEN PL, SCULLY P, SHEW JY, WANG JY AND LEE WH. (1989). Phosphorylation of the retinoblastoma gene product is modulated during the cell cycle and cellular differentiation. *Cell*, **58**, 1193−1198.

DE CAPRIO JA, LUDLOW JW, FIGGE J, SHEW J-Y, HUANG C-M, LEE W-H, MARSILIO E, PAUCHA E AND LIVINGSTON DM. (1988). SV40 large tumour antigen forms a specific complex with the product of the retinoblastoma susceptibility gene. *Cell*, **54**, 275−283.

DYSON N, HOWLEY PM, MÜNGER K AND HARLOW E. (1989). The human papilloma virus-16 E7 oncoprotein is able to bind the retinoblastoma gene product. *Science*, **243**, 934−937.

EGAN D, BAYLEY ST AND BRANTON PE. (1989). Binding of the Rb1 protein to E1A products is required for adenovirus transformation. *Oncogene*, **4**, 383−388.

FIGGE J, BAKST G, WEISHEIT D, SOLIS O & ROSS JS. (1991). Image analysis quantification of immunoreactive retinoblastoma protein in human tyroid neoplasms with a streptavidin–biotin–peroxidase staining technique. *Am. J. Pathol.*, **139**, 1213–1219.

GINSGERG AM, RAFFELD M AND COSSMAN J. (1991). Inactivation of the retinoblastoma gene in human lymphoid neoplasms. *Blood*, **77**, 833–840.

HOROWITZ JM, PARK S-H, BOGENMANN E, GHENG J-C, YANDELL DW, KAYE FJ, MINNA JD, DRYJA TP AND WEINBERG RA. (1990). Frequent inactivation of the retinoblastoma anti-oncogene is restricted to a subset of human tumour cells. *Proc. Natl Acad. Sci. USA*, **87**, 2775–2779.

HOROWITZ JM, YANDELL DW, PARK SH, CANNING S, WHYTE P, BUCHKOVIC K, HARLOW E, WEINBERG RA AND DRYJE TP. (1989). Point mutational inactivation of the retinoblastoma anti-oncogene. *Science*, **243**, 937–940.

ISHIKAWA J, XU H-J, HU S-X, YANDELL DW, MAEDA S, KAMIDONO S, BENEDICT WF AND TAKAHASHI R. (1991). Inactivation of the retinoblastoma gene in human bladder and renal cell carcinomas. *Cancer Res.*, **51**, 5736–5743.

KORNBLAU SM, CHEN N, DEL GIGLIO A, O'BRIEN S, & DEISSEROTH B. (1994). Retinoblastoma protein expression is frequently altered in chronic lymphocytic leukemia. *Cancer Res.*, **54**, 242–246.

LEE WH, BOOKSTEIN RE AND LEE EYHP. (1990). Molecular biology of the human retinoblastoma gene. In *Tumour Suppressor Genes*, Klein G. (ed.). pp. 169–199. Marcel Dekker: New York

LEES JA, BUCHKOVICH KJ, MARSHAK DR, ANDERSON CW AND HARLOW E. (1991). The retinoblastoma protein is phosphorylated on multiple sites by human cdc2. *EMBO J.*, **10**, 4279–4290.

LIN BT-Y, GRUENWALD S, MORLA AO, LEE W-H AND WANG JYJ. (1991). Retinoblastoma cancer suppressor gene product is a substrate of the cell cycle regulator cdc2 kinase. *EMBO J.*, **10**, 857–864.

MARTINEZ JC, PIRIS MA, SANCHEZ-BEATO M, VILLUENDAS R, ORRADRE JL, ALGARA P, SANCHEZ-VERDE L AND MARTINEZ P. (1993) Retinoblastoma (Rb) gene product expression in lymphomas. Correlation with Ki67 growth fraction. *J. Pathol.*, **169**, 405–412.

MARTINEZ JC, MATEO M, SANCHEZ-BEATO M, VILLUENDAS R, ORRADRE JL, ALGARA P, SANCHEZ-VERDE L, GARCIA P, LOPEZ C, MARTINEZ P AND PIRIS MA. (1995). MDM2 expression in lymphoid cells and reactive and neoplastic lymphoid tissue. Comparative study with p53 expression. *J. Pathol.*, **177**, 27–34.

MATEO MS, SANCHEZ-BEATO M, MARTINEZ JC, ORFAO A, ORRADRE JL AND PIRIS MA. (1995). P53, Rb and bcl-2 expression during the cell cycle: a study in phytohaemagglutinin stimulated lymphocytes and microwave irradiated lymphoid tissue sections. *J. Clin. Pathol.*, **48**, 151–159.

MIHARA K, CAO XR, YEN A, CHANDLER S, DRISCOLL B, MURPHREE AL, T'ANG A AND FUNG Y-KT. (1989). Cell cycle-dependent regulation of phosphorylation of the human retinoblastoma gene product. *Science*, **246**, 1300–1303.

SCHOUTEN HC, SANGER WG, DUGGAN M, WEISENBURGER DD, MACLENNAN KA AND ARMITAGE JO. (1989). Chromosomal abnormalities in Hodgkin's disease. *Blood*, **73**, 2149–2154.

SHIMIZU E, COXON A, OTTERSON GA, STEINBERG SM, KRATZKE RA, KIM YW, FEDORKO J, OIE H, JOHNSON BE, MULSHINE JL, MINNA JD, GAZDAR AF AND KAYE FJ. (1994). Rb protein status and clinical correlation from 171 cell lines representing lung cancer, extrapulmonary small cell carcinoma, and mesothelioma. *Oncogene*, **9**, 2441–2448.

SLEBOS RJC, LEE MH, PLUNKETT BS, KESSIS TD, WILLIAMS BO, JACKS T, KASTAN MB AND CHO KR (1994). p53-dependent G1 arrest involves pRb related proteins and is disrupted by the human papillomavirus 16 E7 oncoprotein. *Proc. Natl Acad. Sci. USA*, **91**, 5320–5324.

STILGENBAUER S, DÖNERH, BULGAY-MÖRSCHEL M, WEITZ S, BENTZ M AND LICHTER P. (1993). High frequency of monoallelic retinoblastoma gene deletion in B-cell chronic lymphoid leukemia shown by interphase cytogenetics. *Blood*, **8**, 2118–2124.

SZEKELY L, SELIVANOVA G, MAGNUSSON KP, KLEIN G AND WIMAN KG. (1993). EBNA-5, an Epstein–Barr virus-encoded nuclear antigen, binds to the retinoblastoma and p53 proteins. *Proc. Natl Acad. Sci. USA*, **90**, 5455–5459.

TERADA N, LUCAS JJ AND GELAND EW. (1991). Differential regulation of the tumour suppressor molecules, retinoblastoma susceptibility gene product (Rb) and p53, during cell cycle progression of normal human T cells. *J. Immunol.*, **147**, 698–704.

TILLY H, BASTARD C, DELASTRE T, DUVAL C, BIZET M, LENOMARD B, DAUCE J-P, MONCONDUIT M AND PIGUET H. (1991). Cytogenetic studies in untreated Hodgkin's disease. *Blood*, **77**, 1298–1304.

VENTER DJ, BEVAN KL, LUDWIG RL, RILEY TEW, JAT PS, TOMAS DGT AND NOBLE MD. (1991). Retinoblastoma gene deletions in human glioblastomas. *Oncogene*, **6**, 445–448.

VINDELOV LL, CHRISTENSEN IJ AND NISSEN NI. (1983). A detergent-trypsin method for the preparation of nuclei for flow cytometric DNA analysis. *Cytometry*, **3**, 323–327.

WHITE E. (1994). P53, guardian of Rb. *Nature*, **371**, 21–22.

WHYTE P, BUCHKOVICH JJ, HOROWITZ JM, FRIEND SH, RAYBUCK M, WEINBERG RA AND HARLOW E. (1988). Association between an oncogene and an anti-oncogene: the adenovirus E1A proteins bind to the retinoblastoma gene product. *Nature*, **334**, 124–129.

WILLIAMS BR, REMINGTON L, ALBERT DM, MUKAI S, BRONSON RT AND JACKS T. (1994). Cooperative tumorigenic effects of germline mutations in Rb and p53. *Nature Genet.*, **7**, 480–484.

XIAO Z-X, CHEN J, LEVINE AJ, MODJTAHEDI N, XING J, SELLERS WR AND LIVINGSTON DM. (1995). Interaction between the retinoblastoma protein and the oncoprotein MDM2. *Nature*, **375**, 694–697.

XU H-J, HU S-X, CAGLE PT, MOORE GE AND BENEDICT WF. (1991*a*). Absence of retinoblastoma protein expression in primary non-small cell lung carcinomas. *Cancer Res.*, **51**, 2735–2739.

XU H-J, XU S-X AND BENEDICT WF. (1991*b*). Lack of nuclear Rb protein staining in G0/middle G1 cells: correlation to changes in total Rb protein level. *Oncogene*, **6**, 1139–1146.